# Analysis of miRNA expression profile induced by short term starvation in breast cancer cells treated with doxorubicin

**DOI:** 10.18632/oncotarget.18028

**Published:** 2017-05-19

**Authors:** Sergio Rizzo, Antonina Cangemi, Antonio Galvano, Daniele Fanale, Silvio Buscemi, Marcello Ciaccio, Antonio Russo, Sergio Castorina, Viviana Bazan

**Affiliations:** ^1^ Department of Surgical, Oncological and Oral Sciences, Section of Medical Oncology, University of Palermo, Palermo, Italy; ^2^ Department of Internal and Specialistic Medicine (DIBIMIS), Laboratory of Clinical Nutrition, University of Palermo, Palermo, Italy; ^3^ Section of Clinical Biochemistry and Clinical Molecular Medicine, Department of Biopathology and Medical Biotechnology, University of Palermo, U.O.C. Laboratory Medicine, Policlinico University Hospital, Palermo, Italy; ^4^ Fondazione Mediterranea “G.B. Morgagni”, Catania, Italy; ^5^ Department of Biomedical and Biotechnological Sciences, University of Catania, Catania, Italy

**Keywords:** chemotherapy response, doxorubicin, microRNAs, short term starvation, triple negative breast cancer cells

## Abstract

Recent studies showed that dietary approaches restricting food intake can be helpful to hinder tumor progression. To date, the molecular mechanisms are unclear and a key role seems to be exerted by nutrient-related signaling pathways. Since several evidences showed that non-coding small RNAs, including microRNAs, are correlated to cancer progression and antiblastic treatment response, our work aims to study their involvement in a triple negative breast cancer (TNBC) cell line treated with doxorubicin under Short Term Starvation (STS) condition.

Human TNBC cell line MDA-MB-231 and healthy breast cell line MCF10A were treated with 1 μM doxorubicin for 24 h under STS condition for 48 h and miRNA expression profiles were analyzed using Taqman^®^ Low Density Array A human microRNA microfluidic cards. In addition, the expression of specific mRNAs and miRNAs differentially expressed under STS was analyzed using Real-time PCR analyses.

MiRNA expression profile analysis in MDA-MB-231 and MCF10A cells treated with doxorubicin under STS for 48 h could explain the molecular mechanisms underlying anticancer effects associated to STS. Among deregulated miRNAs, a subset, including miR-15b, miR-23a, miR-26a, miR-29a, miR-106b, miR-128, miR-149, miR-181a, miR-192, miR-193b, miR-195, miR-324-3p and miR-494, has been shown to be involved in pathways related to drug sensitivity/resistance.

The obtained data from our study suggest a potential involvement of some miRNAs in molecular pathways mediating the anticancer effects of STS in doxorubicin-treated breast cancer cells. Preliminary results seem to be encouraging and, in future, could allow the discovery of new potential targets useful for the development of new therapeutic approaches.

## INTRODUCTION

Chemotherapy represents the main strategy for the treatment of various types of cancer but it is often accompanied by side effects whose severity can lead to reduce drug effectiveness or interrupt therapy especially in aggressive tumors such as triple negative breast cancer (TNBC) [1].

TNBC is a breast cancer (BC) subtype characterized by lack of progesterone receptor (PgR), estrogen receptor (ER), and human epidermal growth factor receptor 2 (HER-2) expression. This explains why, to date, chemotherapy remains the main therapeutic option in TNBC [2, 3].

Currently, several researchers are working and focusing their attention to find a system that makes chemotherapy more tolerable avoiding that treatment compromises the viability of healthy cells. Encouraging data are provided by various approaches that modulate food intake producing antimutagenic, antibacterial, and anticarcinogenic effects and reducing reactive oxygen species (ROS) and inflammatory cytokines. Since adipose tissue may be also involved in BC carcinogenesis by producing pro-inflammatory cytokines, including IL-6 and TNF-α, ROS and matrix metalloproteases, dietary restriction-mediated changes in cancer-associated adipocyte (CAA) sizes may regulate the tumor microenvironment functions resulting in reduction in adipokine secretion [4, 5]. There are several types of dietary restrictions (DR), differentiated according to the ingested nutrients (such as protein or calorie restrictions) or fasting period length (short or long term starvation), that, without distinction, increase mammalian life-span and have a protective role against age-related effects [6]. Among them, Short Term Starvation (STS) is the most feasible approach for patients already inclined to weight loss because of cancer itself or chemotherapy [7], as it consists of an alternation of short fasting cycles and periods not subjected to dietary restrictions [8].

Raffaghello et *al*. [9] evaluated the influence of high-dose chemotherapy and STS in murine models testing the manifestation of side effects after the administration of Etoposide, a drug with a non-specific toxicity profile, in a group of mice undergone to 48 h STS and a control group fed ad libitum. Unlike the fasting mice, control group showed side effects and signs of pain and stress. STS affected also mice vitality. In fact, the mortality rate of fed group was around 43%, while the acute toxicity of Etoposide caused only one dead in fasting group. Moreover, an Etoposide dose four or five times higher than the recommended one for human in presence of 48 h and 60 h STS induced death or toxicity in the control group, while in the starved group mice lost 40% of their weight (recovered after a week of re-feeding). STS effects gave benefits also in ten human volunteers affected by different type of tumors starved for 48–140 h before chemotherapy and 56 h after treatment [9].

Currently there are not studies that identified a single gene or molecular mechanism able to explain STS benefits, but some nutrient-related signaling pathways such as insulin-like growth factor 1 (IGF-1) and its downstream effectors, including c-Jun N-terminal kinase (JNK), extracellular signal-regulated kinase (ERK), p38 mitogen-activated protein kinase (MAPK), and phosphoinositide 3-kinase (PI3K), which are known to regulate several detoxification enzymes, seem to be involved [10].

Recent studies showed that non-coding small RNAs called microRNAs (miRNAs), negatively regulating gene expression via binding to their mRNA target, are involved in different physiological and pathological mechanisms. Their expression has been found to be aberrant in many cancer types where miRNAs can act as tumor suppressor (TS-miRNAs), able to facilitate cancer cell death and/or inhibit cancer cell growth, or oncogenes (oncomiRs), that promote cancer progression and angiogenesis [11]. A significant number of genes encoding miRNAs is located in genomic regions frequently altered in tumors such as fragile sites, deleted or amplified regions and common areas of break-point, providing further evidence of their role in cancer pathogenesis [12, 13].

MiRNAs could be considered therapeutic targets for a large variety of age-related diseases and it is spreading the idea to use them as a new clinical tool to monitor the progression, prognosis, diagnosis, and treatment responses [14].

Since recent studies demonstrated that miRNAs may regulate sensibility/resistance to antiblastic therapy [15], the aim of this study is to evaluate in deep the molecular changes caused by STS in TNBC cells treated with Doxorubicin focusing our attention on their involvement in molecular pathways mediating the anticancer effects.

## RESULTS

### Short term starvation reduces MDA-MB-231 cell proliferation and viability

*In vitro* cell vitality assays were previously carried out on MDA-MB-231 cells using three different concentrations of Doxorubicin (1, 2.5 and 5 μM) at three different time-points (6, 12 and 24 h) in order to identify the lower dose (1 μM) and best time-point (24 h) sufficient to induce an anti-proliferative effect on MDA-MB-231 cells (data not shown).

Further viability assays showed that MDA-MB-231 cells subjected to STS were less vital than tumor cells in standard medium (NoSTS). We observed that a STS condition maintained for 48 h induced a progressive and significant reduction in cell viability. The association of STS and treatment with 1 μM Doxorubicin for 24 h further reduced MDA-MB-231 growth.

**Figure 1:**
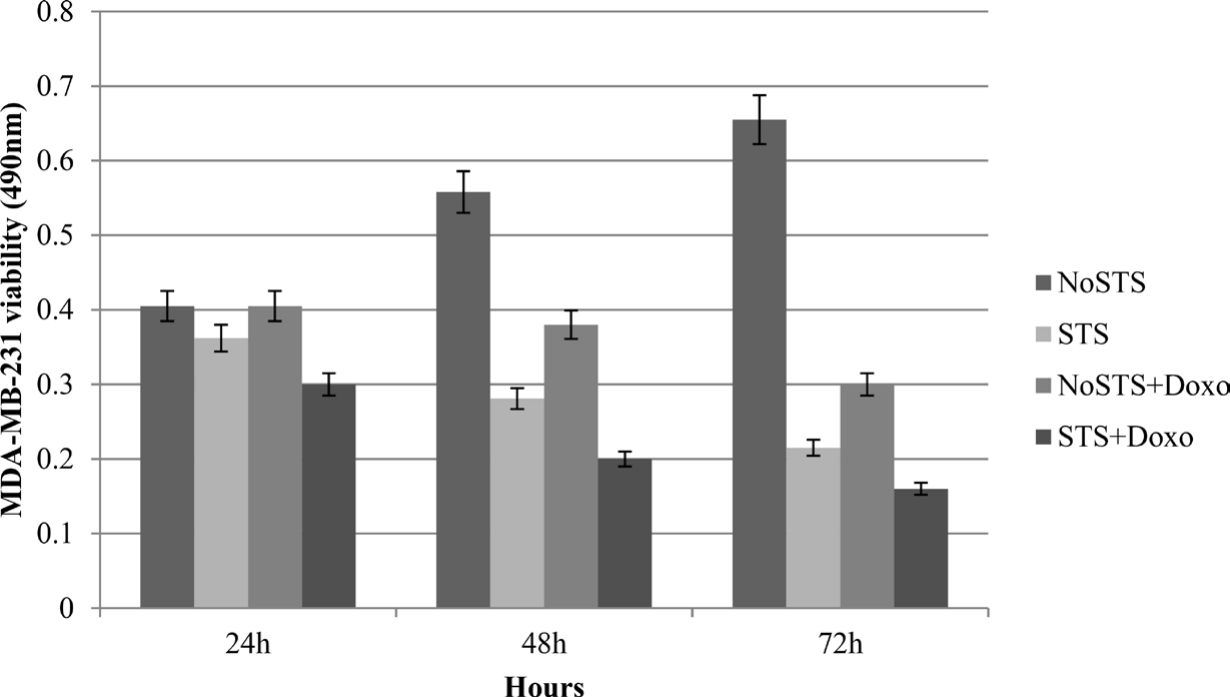
Vitality assays in MDA-MB-231 cells after 24 h, 48 h and 72 h of STS with and without 1 μM Doxorubicin treatment for 24 h

**Figure 2:**
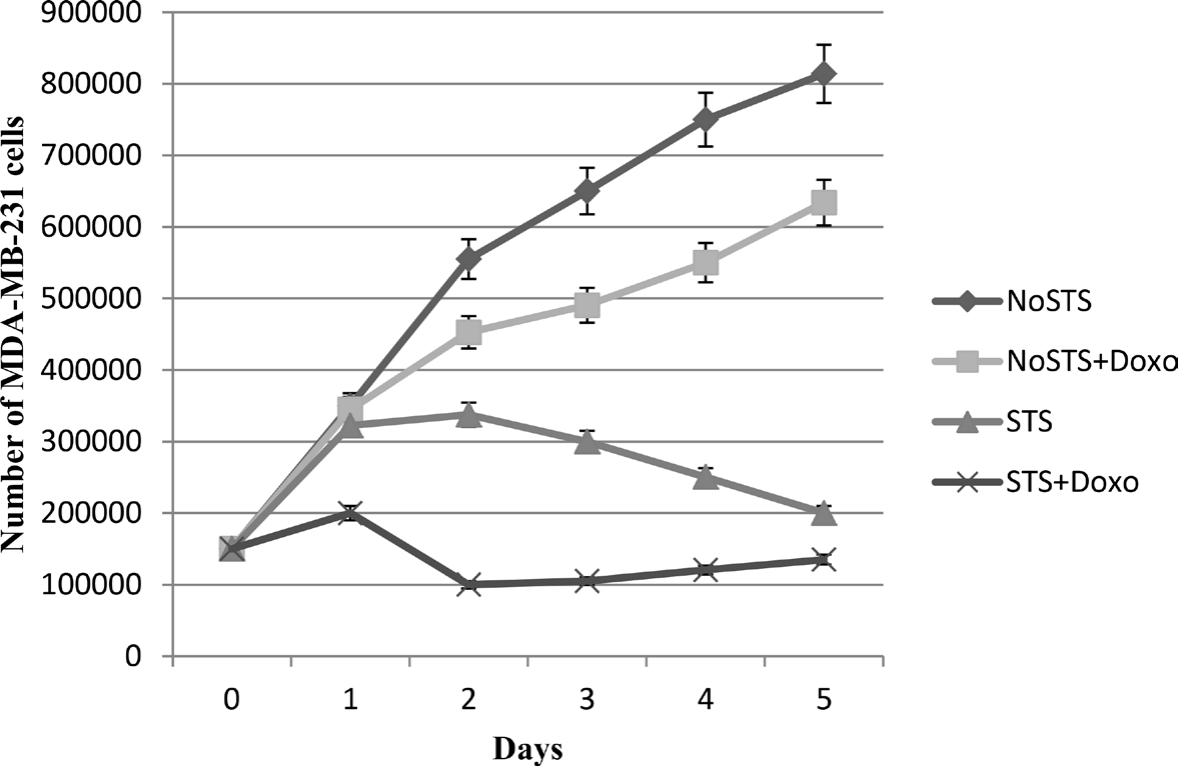
Growth curves of MDA-MB-231 cells after 1, 2, 3, 4 and 5 days of STS with and without 1 μM Doxorubicin treatment for 24 h

In addition, we observed that STS causes a reduction in MDA-MB-231 cell viability more evident after chemotherapeutic treatment (Figure 1). Consistent results were obtained by analysis of proliferation curves. During five days of STS, a progressive proportional decrease in MDA-MB-231 viability was observed. Doxorubicin treatment induced a cell proliferation reduction especially remarkable in MDA-MB-231 cells previously subjected to STS (Figure 2). Therefore, the greatest effect seems to occur when STS is associated with chemotherapy.

### Short term starvation protects MCF10A cells from chemotherapeutic treatment

**Figure 3:**
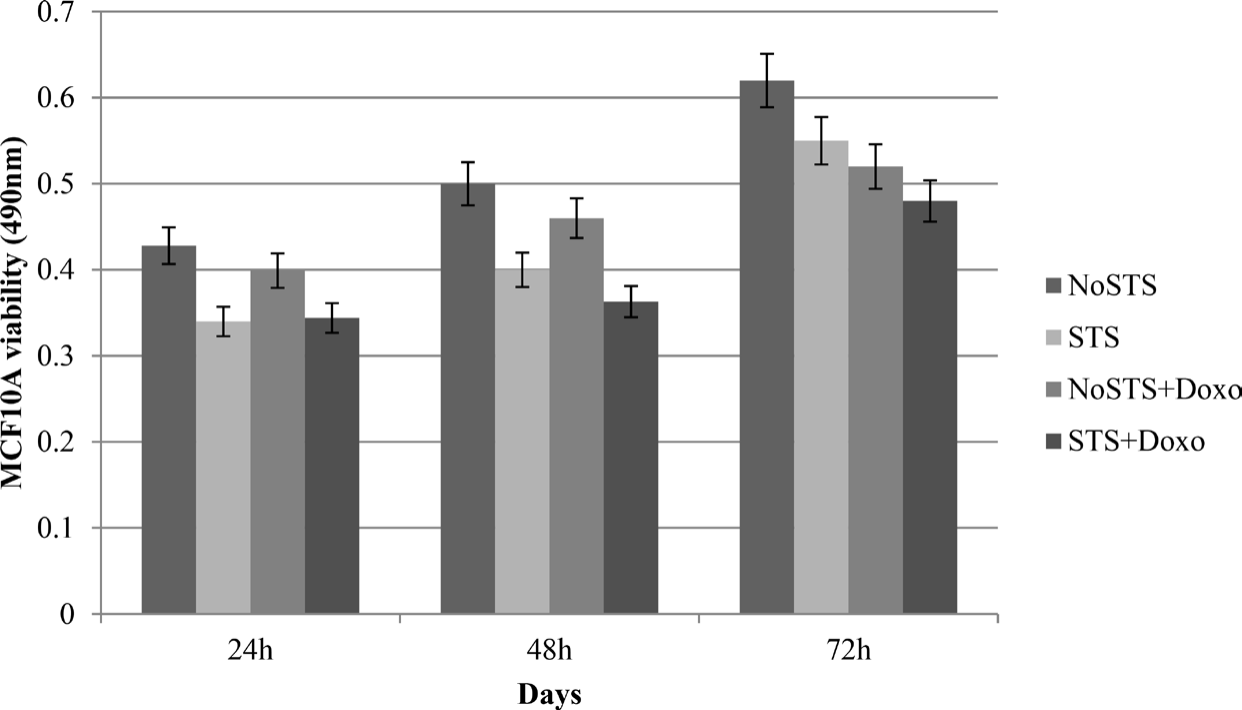
MCF10A cell viability after 24 h, 48 h and 72 h of STS with and without 1 μM Doxorubicin treatment for 24 h

**Figure 4:**
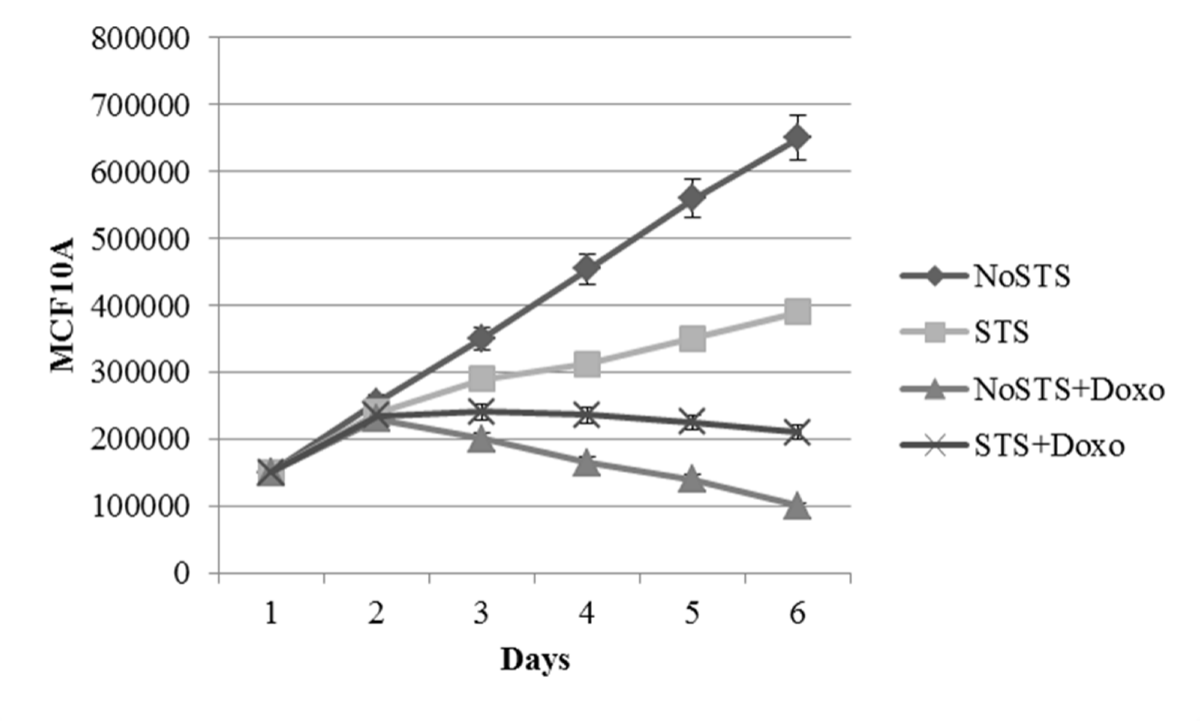
Growth curves of MCF10A cells after 1, 2, 3, 4 and 5 days of STS with and without 1 μM Doxorubicin treatment for 24 h

STS caused a non-significant reduction in cell viability of healthy cells (MCF10A). Interestingly, it was observed that STS associated chemotherapeutic treatment for 24 h with 1 μM Doxorubicin seems to protect MCF10A cells (Figure 3). Effects of STS on MCF10A cells has been confirmed through the analysis of growth curves (Figure 4). Therefore, STS induces a reduction in MCF10A cell viability lower than that observed in MDA-MB-231 cells and seems to have a protective role in presence of chemotherapy.

### MiRNA expression profile induced by STS in MDA-MB-231 cells

Since several experimental evidences showed that miRNAs may be important diagnostic and prognostic biomarkers in cancer, alterations in miRNA expression patterns may be correlated with specific pathological conditions, clinical outcome and therapy response [16–21]. In order to understand the effects of STS on miRNA expression profile, we performed a large-scale analysis of 377 miRNAs on MDA-MB-231 cells treated with 1 μM doxorubicin for 24 h compared to untreated cells. This microarray analysis showed that approximately 23% of miRNAs was significantly deregulated in MDA-MB-231 cells subjected to STS for 48 h and treated with 1 μM Doxorubicin for 24h (Supplementary Figure 1), and, among these, 61.8% of them was downregulated whereas 38.2% was upregulated (Supplementary Figure 2). Moreover, about 4.5% of miRNAs, including miR-148a, miR-154, miR-194, miR-361-5p and miR-449b, was specifically expressed only in treated MDA-MB-231 cells undergone to STS (Supplementary Figure 3). MiRNA expression assays by quantitative real-time PCR analysis have confirmed the previously obtained results concerning the expression of miR-148a (Hs04273238_s1) and miR-154 (Hs04231525_s1) (Supplementary Figure 4).

At the same time, since *CCND1* has been shown to be a hypothetical gene target for miR-449b and miR-194, whereas *VEGFA* for miR-361-5p, we have analyzed also the expression levels of *CCND1* (Hs00765553_m1) and *VEGFA* (Hs00900055_m1) mRNAs in MDA-MB-231 cells subjected to the same conditions, in order to confirm the coherence of our results (Supplementary Figure 5).

**Figure 5:**
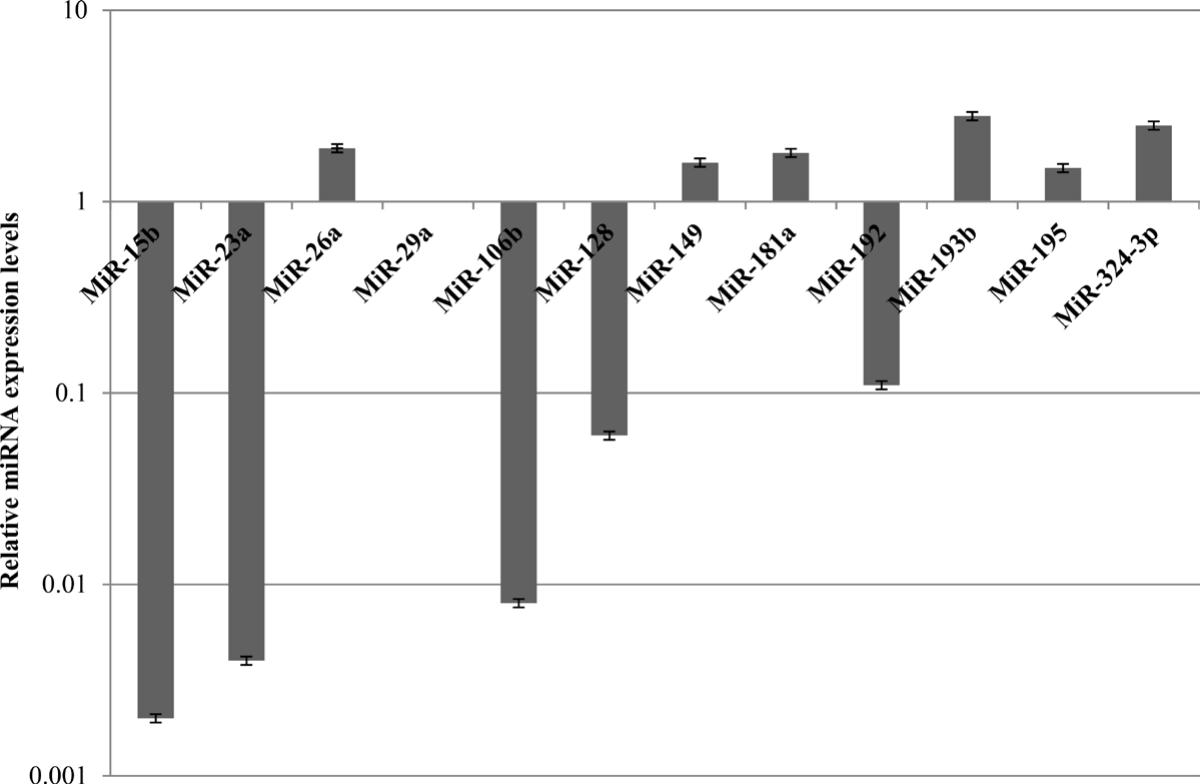
Expression levels of miRNAs involved in chemotherapy response in MDA-MB-231 after 48 h of STS and 1 μM Doxorubicin treatment for 24 h

Since miR-26a, miR-149, miR-181a, miR-193b, miR-195 and miR-324-3p are resulted to be upregulated (Figure 5) and data in literature showed their involvement in increasing drug sensitivity, we assessed also the expression levels of miR-26a and its potential target *E2F2* (Hs00918090_m1) (Supplementary Figure 5). The upregulation of other miRNAs, such as miR-149 (Hs04231523_s1), miR-181a (Hs04231460_s1), miR-193b (Hs04231607_s1), miR-195 (Hs03656088_s1) and miR-324-3p (Hs04273262_s1), was confirmed by TaqMan microRNA expression assays (Supplementary Figure 4).

Since miR-15b, miR-23a, miR-29a, miR-106b, miR-128, miR-192 and miR-494 were found downregulated and have been shown to induce chemoresistance (Figure 5), we evaluated the expression of some of them, including miR-15b (Hs04231486_s1), miR-23a (Hs03659093_s1) and miR-29a (Hs03849009_s1), through TaqMan microRNA expression assays (Supplementary Figure 4). Finally, given that *PTEN* has been shown to be a hypothetical gene target for miR106b and miR-494, *BAX* for miR-128, and *BIM* (or *BCL2L11*) for miR-192, their gene expression was analyzed by means of quantitative Real-time PCR analysis (Supplementary Figure 5).

### STS-induced MiRNA expression analysis in MCF10A cells

**Figure 6:**
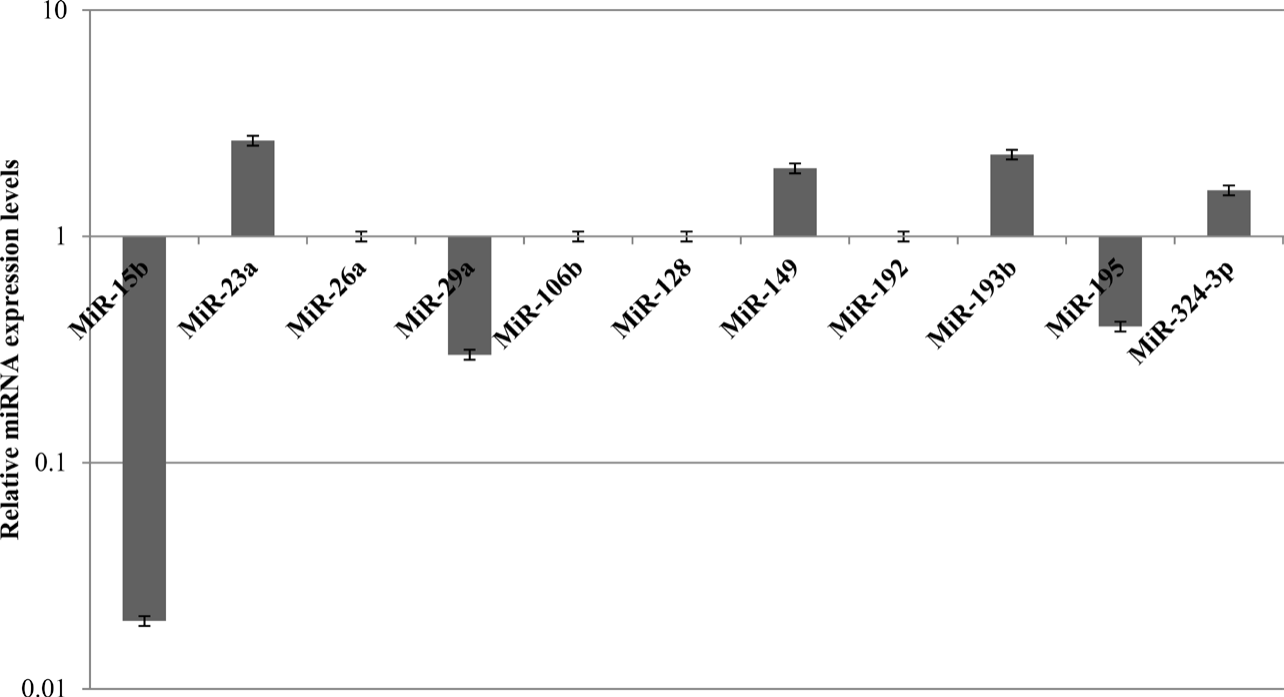
Expression levels of MiRNAs involved in chemotherapy response in MCF10A cells after 48 h of STS and 1 μM Doxorubicin treatment for 24 h

The expression of miRNAs involved in chemotherapy response was also evaluated in MCF10A cells treated with 1 μM doxorubicin and subjected to STS for 48 h. Unlike MDA-MB-231 cells, it was observed an heterogeneous miRNAs expression profile in healthy cells. Among miRNAs involved in chemotherapy response, miR-26a, miR-106b, miR-128 and miR-192 were not found significantly deregulated. Conversely, miR-23a, miR-149, miR-193b, and miR-324-3p were upregulated, whereas miR-15b, miR-29a, miR-181a, miR-195, and miR-494 were downregulated (Figure 6).

MiRNAs deregulation was confirmed by TaqMan miRNA expression assays that specifically allowed to detect the expression levels of following miRNAs: miR-23a (Hs03659093_s1), miR-149 (Hs04231523_s1), miR-193b (Hs04231607_s1), miR-324-3p (Hs04273262_s1), miR-15b (Hs04231486_s1), miR-29a (Hs03849009_s1), miR-181a (Hs04231460_s1), and miR-195 (Hs03656088_s1) (Supplementary Figure 6).

Additionally, since *PTEN* has been shown to be one of hypothetical gene targets for miR-494, we analyzed its expression in MCF-10A cells treated with doxorubicin and undergone to STS, confirming the consistency of previously obtained results (Supplementary Figure 7).

## DISCUSSION

Chemotherapy exhibits a cytotoxic profile that affects both cancer and healthy cells leading to worsening side effects that compromise treatment efficacy. It could be necessary to design a strategy in order to focus antiblastic drug activity against tumor mass. A non-invasive tool able to make chemotherapy more suitable seems represented by STS but the basic mechanisms are unclear. Since studies indicated miRNAs as key factors in different pathological and physiological process, this manuscript aims to investigate their involvement in triple negative breast cancer cell line MDA-MB-231 and healthy mammary gland MCF10A cells after 48 h of STS and 24 h of 1 μM Doxorubicin treatment. Our team focused the attention on those miRNAs that modulate chemotherapeutic response obtaining encouraging preliminary data. In MDA-MB-231 cells, among upregulated miRNAs there are miR-26a, miR-149, miR-181a, miR-193b, miR-195 and miR-324-3p that increase drug responsiveness. In particular, previous studies showed that miR-26a sensitized gastric cancer to cisplatin targeting NRAS and E_2_F_2_ [22], miR-149 increased chemosensitivity of glioblastoma to Temozolomide treatment through a RAP1B-mediated remodeling of cellular cytoskeleton [23], miR-181a enhanced Adryamicin-induced apoptosis targeting Bcl-2 [24], miR-193b sensitized cancer cells to Doxorubicin targeting myeloid cell leukemia-1 (MCL-1) [25], miR-195 increased Adriamycin sensibility by downregulating RAF [26], and, finally, miR-324-3p induced drug sensitivity reducing its SMAD7 target mRNA that is associated with lung, pancreas and skin cancer [27].

We found that some miRNAs, including miR-15b, miR-23a, miR-29a, miR-106b, miR-128, miR-192 and miR-494, were downregulated in MDA-MB-231 cells under STS conditions. MiR-15b and miR-23a have been shown to increase Cisplatin-resistance in lung cancer cell line A549 [28] and in tongue squamous cell carcinoma [29], whereas miR-29a induced Adriamycin and Docetaxel resistance in breast cancer (BC) [30], miR-128 enhanced antiblastic resistance in BC cells targeting *BAX* [31], miR-192 promoted Cisplatin-resistance in lung cancer cells A549/DDP [32], and, finally, miR-106b and miR-494 conferred radioresistance and Sorafenib-resistance in colorectal cancer and hepatocellular carcinoma silencing PTEN and p21 [33–35].

Unlike MDA-MB-231 cells, miRNAs involved in antiblastic response in MCF10A cells were heterogeneously expressed and this could explain why healthy cells under STS seem to be more resistant to Doxorubicin treatment.

Considering the multifunctional nature of miRNAs, further investigations are needed to confirm the obtained results. Preliminary analysis could represent an important step toward the comprehension of STS-inducted molecular changes and improvement of clinical strategies. Pharmacologically active molecules could be developed in order to silence an oncomiR or increase expression of tumor suppressor miRNAs. Understanding mechanisms underlying miRNA role in the cancer development and progression could provide an useful tool to develop therapeutic strategies able to adopt miRNAs as potential targets for cancer treatment.

## MATERIALS AND METHODS

### Cell cultures

*In vitro* analyses were performed on a triple negative breast cancer cell line MDA-MB-231 and healthy mammary gland cell line MCF10A. Both cell lines were grown at 37°C, 5% CO_2_ and 80% confluence. Maintenance medium for MDA-MB-231 cells was DMEM-GlutaMax (4.5gr D-Glucose/Liter, ThermoFisher) enriched with Fetal Bovine Serum (FBS-10%), Non Essential Amino Acids (NEAA-1%) and Streptomycin-Penicillin (Strepto/Pen). Maintenance medium for MCF10A cells was DMEM F:12 enriched with MEGM™ Mammary Epithelial Cell Growth Medium (Lonza), 1gr/L Glucose and the 10% FBS. For STS experiments DMEM glucose-free (ThermoFisher) enriched with 0.5 gr/L Glucose, 1% FBS, 1% NEAA, 1% Strepto/Pen and 25 mM Hepes was used.

Cells were treated with 1 μM Doxorubicin for 24 h and STS conditions were maintained at different time points (24, 48 and 72 hours).

### Cell proliferation and viability analysis

The cell counting was performed by means of a Burker chamber, a device of manual counting of cells at the electron microscope. Both cell groups were subjected to 1 μM Doxorubicin for 24 h. Cells were counted at different time-points of fasting in order to evaluate the most suitable STS condition.

Cell viability was assessed through Trypan blue and MTT colorimetric assays. The first is based on the principle that dead cells, having a damaged cell membrane, internalize the dye and become blue. Both dead and living cells are counted by Burker Chamber.

MTT colorimetric assay was performed starving 8000 cells in a 96-well plate and subjecting them to fasting and chemotherapy treatment. The assay was performed using the MTT Cell Growth kit (Millipore) consisting of 3-(4,5-dimethylthiazol-2)-2,5-difeniltetrazolium bromide. It measures the mitochondrial enzyme activity that reduce tetrazolium salts to formazan. The coloration intensity of the obtained solution is directly proportional to cell viability.

### MiRNA expression profile analysis

Total cellular RNA and miRNAs were isolated using the miRNeasy Mini Kit (Qiagen Inc, Valencia, CA). The quality of samples was controlled through RNA 6000 Nano Assay (Agilent Techologies, Palo Alto, CA, USA) using 2100 Bioanalyzer (Agilent Technologies, Santa Clara, CA) and quantified through the spectrophotometer NanoDrop ND-1000 (CELBIO). To analyze miRNA expression profile, we used TaqMan® Low Density Array A Human MicroRNA v2.0 (Life Technologies, Carlsbad, California, USA). The arrays were processed and analyzed in accordance to manufacturer's protocols as previously described [36, 37]. For each experimental condition two biological replicates were used. The data were normalized using the RNU48 as endogenous control. The 2^−ΔΔCT^ (delta-delta-Ct algorithm) method was used to evaluate the relative changes in miRNA expression. A miRNA was considered differentially expressed when estimated *P*-value was < 0.05. As regards the differentially expressed miRNAs we established a cut off of fold change > 1.5 for up-regulated miRNAs and < 0.5 for down-regulated miRNAs.

### Quantitative real-time PCR analysis

Ten ng of total RNA from other independent samples were reverse transcribed using Taqman MicroRNA Reverse Transcription Kit (Life Technologies, Carlsbad, California, USA) according to manufacturer's instructions. cDNA was amplified using the following Taqman MicroRNA assays: hsa-miR-15b, hsa-miR-23a, hsa-miR-29a, hsa-miR-148a, has-miR-149, hsa-miR-154, hsa-miR-181a, hsa-miR-193b, hsa-miR-195, hsa-miR-324-3p (Life Technologies, Carlsbad, California, USA). The reactions were performed in triplicate and changes in the target miRNA content relative to RNU6B were determined using the comparative Ct method to calculate changes in Ct, and, ultimately, fold and percent change. Undetermined values of Ct were estimated as 40 Ct (the last cycle of the reactions), whereas a cut off < 35 Ct was used to select reliably quantifiable miRNAs.

For gene expression analysis, 2 μg of total RNA were reverse transcribed into single-stranded cDNA using High Capacity cDNA Reverse Transcription Kit (Applied Biosystems, Foster City, CA, USA) according to the vendor's instructions, as previously described [38, 39]. cDNA was amplified using the following Taqman gene expression assays (Applied Biosystems): Hs00765553_m1 for CCND1; Hs00900055_m1 for VEGFA; Hs00918090_m1 for E2F2; Hs02621230_s1 for PTEN; Hs00180269_m1 for BAX; and Hs00708019_s1 for BIM (or BCL2L11).

Quantitative real-time PCR (qRT-PCR) was carried out with the ABI PRISM 7900 sequence detection system (Applied Biosystems, Foster City, CA, USA) using SDS software version 2.1. The reactions were performed in triplicate and results were normalized using Human β-actin Predeveloped TaqMan assay reagents (Applied Biosystems).

### Data analysis

Data are represented as mean ± S.D (standard deviation). Statistical analyses were performed by Student's *t*-test. Values of *P* < 0.05 were estimated to be statistically significant.

Heat map analysis was performed using the MultiExperiment Viewer (MeV v4.8) program of TM4 Microarray Software Suite. Information concerning MiRNA, mRNA target and related pathways was obtained from the literature and miRBase and Targetscan databases [40].

## SUPPLEMENTARY MATERIALS FIGURES


