# Supplementary material for: Analysis of miRNA expression profile induced by short term starvation in breast cancer cells treated with doxorubicin
Source: Oncotarget. 2017 May 19;8(42):71924–32. doi: 10.18632/oncotarget.18028 (PMC5641100; doi:10.18632/oncotarget.18028)
Supplement: Supplementary file 1 [file oncotarget-08-71924-s001.pdf]

Analysis of miRNA expression profile induced by short term starvation in breast cancer cells treated with doxorubicin

SUPPLEMENTARY MATERIALS

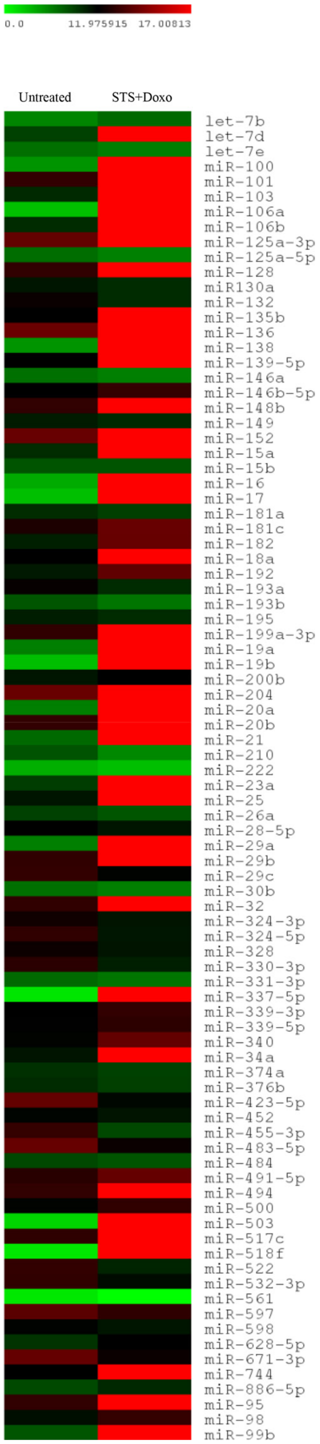

Supplementary Figure 1: Heat map of differentially expressed miRNAs in MDA-MB-231 cells after 48 h of STS and 1 µM Doxorubicin treatment for 24 h.

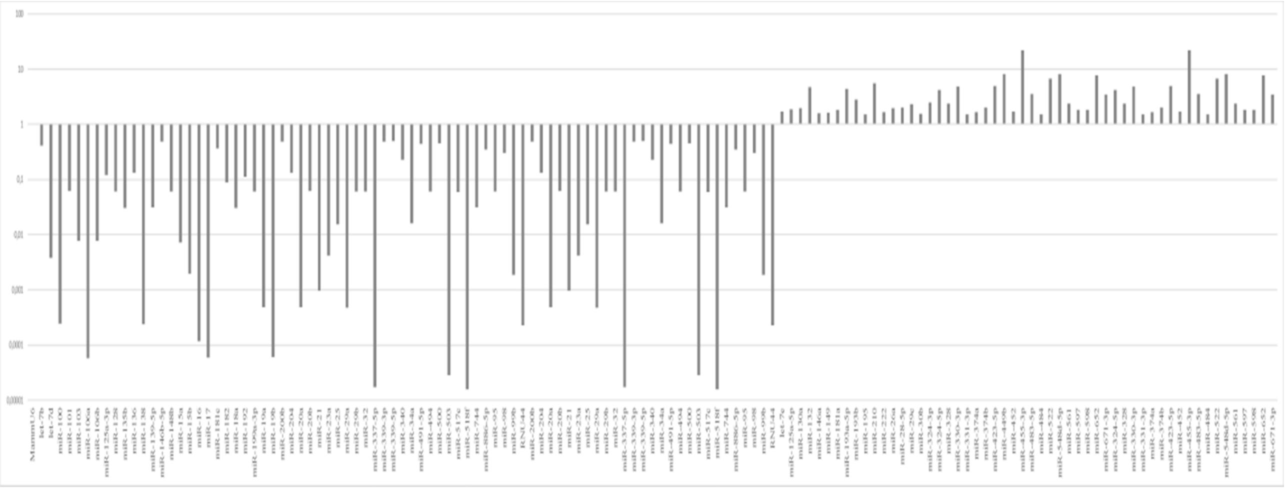

Supplementary Figure 2: Deregulated miRNAs in MDA-MB-231 cells after 48 h of STS and 1 µM Doxorubicin treatment for 24 h.

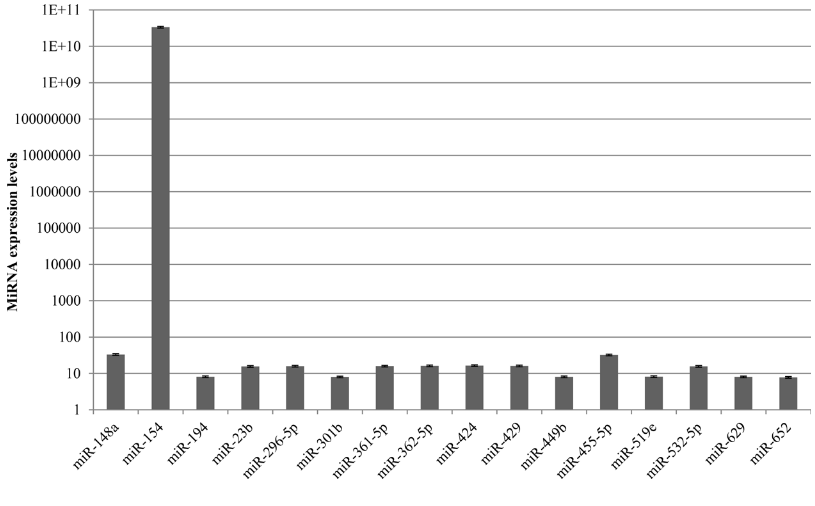

Supplementary Figure 3: MiRNA specifically expressed in MDA-MB-231 cells undergone to STS for 48 h and 1 µM Doxorubicin treatment for 24 h.

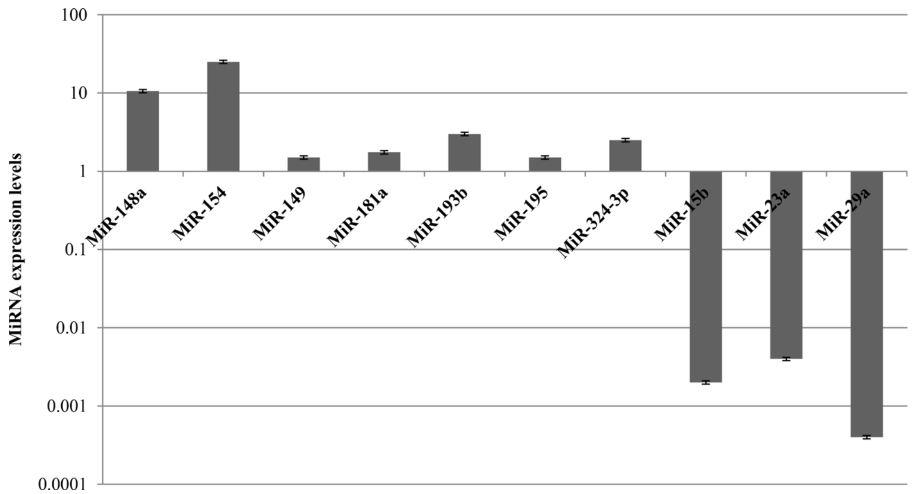

Supplementary Figure 4: Validation of miRNA array data in MDA-MB-231 cells by quantitative real-time PCR analysis.

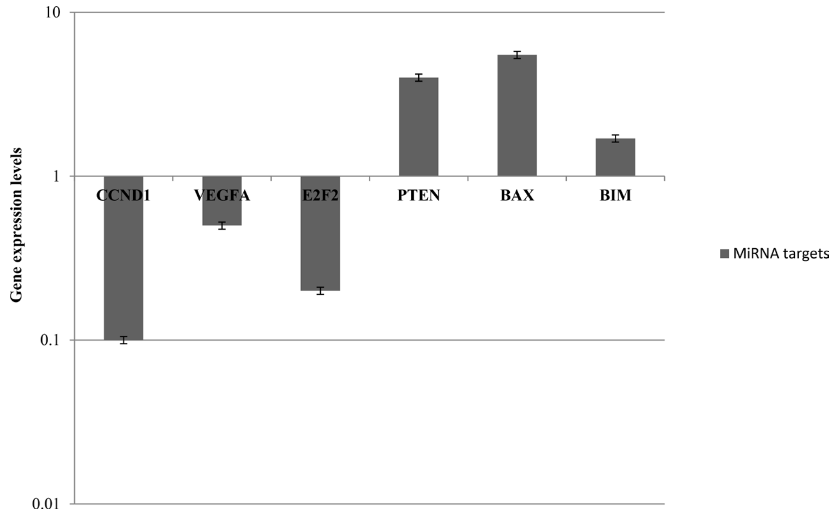

Supplementary Figure 5: Analysis of expression levels of *CCND1*, *VEGFA*, *E2F2*, *PTEN*, *BAX*, *BIM* in MDA-MB-231 cells after 48 h of STS and 1 µM Doxorubicin treatment for 24 h.

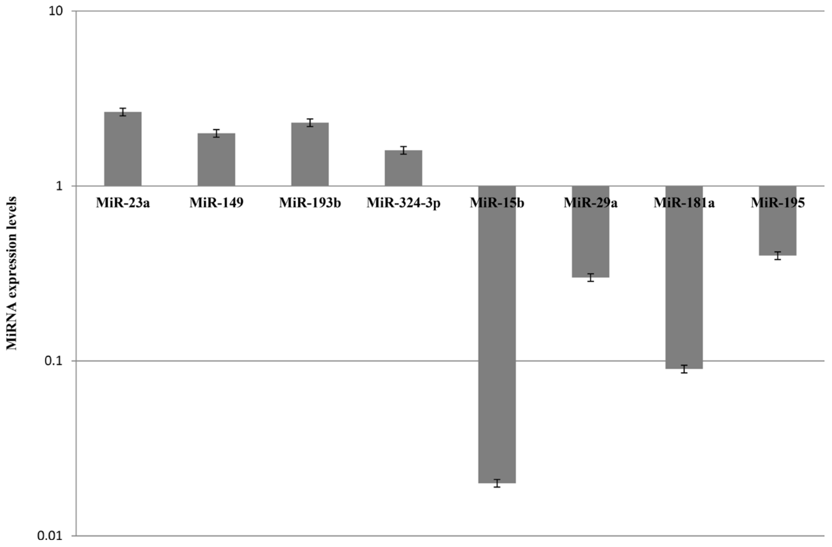

Supplementary Figure 6: Validation of miRNA array data in MCF-10A cells by quantitative real-time PCR analysis.

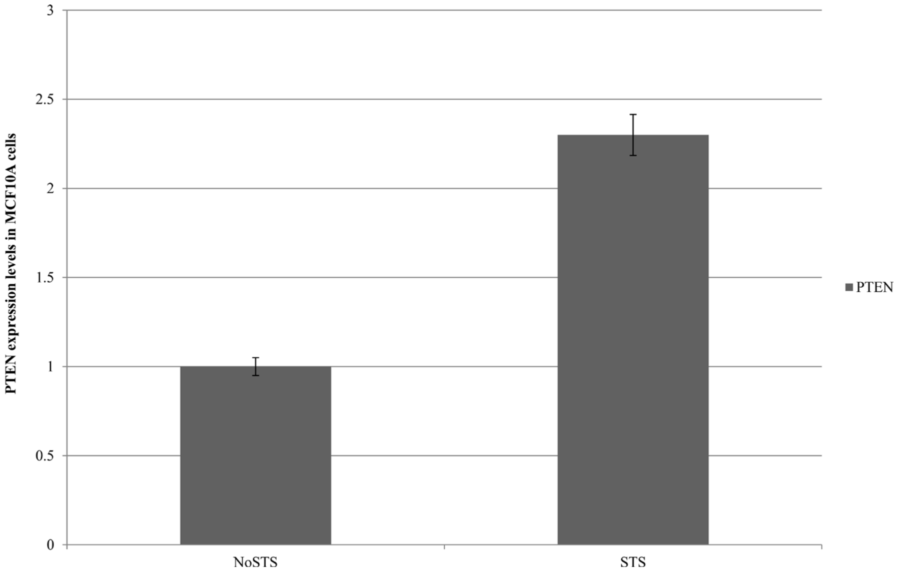

Supplementary Figure 7: PTEN expression levels in MCF-10A cells subjected to STS for 48 h and treated with 1 µM Doxorubicin for 24 h.
